# Students of the Bone Eaters: An Exploration of Osteoclast Multinucleation

**DOI:** 10.1007/s11914-026-00994-x

**Published:** 2026-11-04

**Authors:** Gwen N. Wagner, Morteza Abyadeh, Jacob J. Berger, Jarred M. Whitlock

**Affiliations:** Department of Molecular Physiology and Biological Physics, University of Virginia School of Medicine, Charlottesville, VA USA

## Abstract

**Purpose of Review:**

Here, we explore how the osteoclast’s many nuclei inform behavior, set activity, and underpin osteoclast-driven pathologies.

**Recent Findings:**

Over the last decade, new discoveries have demonstrated that osteoclast multinucleation is a major determinant of activity and driver of pathologic behavior. We open with a review of postnatal skeletal maintenance and the role osteoclasts play in coordinating the multicellular unit responsible for life-long skeletal development. Then, we focus the second half on recent developments in the understanding of how multinucleation influences osteoclast resorptive behavior, underpins skeletal pathology, and close with a discussion on the fate and nature of these syncytial cells. Much of this latter discussion is stimulated by recent observations focusing on the behavior and fate of individual osteoclasts as compared to previous systemic measurements of their activity.

**Summary:**

In the end, work over the last decade - in particular, fate tracing, advances in the live imaging of osteoclast activity, and mechanistic observations of syncytial fission - pushes us to consider the possibility that osteoclasts are more than individual cells but instead operate as community of individuals held in a common boarder. It seems to us that Evolution, when faced with a task too great for one, answered with many - A Syncytial Community.

## Introduction

*“A bit of madness is key, to give us new colors to see. Who knows where it will lead us, and that’s why they need us! So, bring on the rebels, the ripples from pebbles, the friends of cells, wranglers of mice, and tamers of bone. *

*And here’s to the Fools who dream, crazy as we may seem. Here’s to the questions keeping us awake. Here’s to the lovely mess we make.”*

*- adapted from La La Land 2016*

## Life-long Skeletal Remodeling

The Skeleton - one of nature’s great cathedrals - like all magnificent structures must be fashioned and maintained by scores of architects. Much has been garnered about this cathedral’s fashioning during embryogenesis, early postnatal development, and the attentive care exercised in life-long skeletal development - the continual maintenance process once the cathedral’s many beams and arches (*i.e.*, bones) have ceased growing. If you are not an osteologist, or as some colloquially call themselves “a bonehead”, do not worry. One can quickly learn their stories of how bony cathedrals are built [1, 2] and maintained [3, 4] until laid down in final form. In succinct fashion, the skeleton relies on Builders - osteoblasts (**Blasts**), Eaters - osteoclasts (**Clasts**), and Watchers - osteocytes (**Cytes**) of bone. Each are the closest of partners, and they take turns leading one another in cathedral building. In early development, the Blasts build out from *primary ossification centers* within the center of cartilaginous templates - embryonic precursors of bone destined to form the appendicular and axial skeleton - fashioned by chondroblasts (*i.e.*, osteogenesis or ossification). These Builders continue along towards the template’s epiphyses. At these epiphyses, Blasts continue to collaborate with chondroblasts to form growth plates, which facilitate the gradual extension of long bones until shortly after puberty. Alternatively, Blasts also build some flatter, intramembranous bones via intramembranous ossification sans templates (*e.g.*, the craniofacial system) [5]. As Blasts build, Clasts form from precursors carried to ossification sites via angiogenic routes and eat away the substrate to shape biomechanically sound support organs termed bones [6]. As Blasts build, they burry some of their cohort, which further differentiate and remain as entombed Watchers - Cytes. These sentinels build interconnected micro-tunnels that permit their coordination. In analogy, these Watchers function as a sort of contemplative community continually concerned with the cathedral’s form, biomechanics, and function. And so, a living cathedral is built, led by the Blasts.

As vertebrates begin with their methods of locomotion, entombed Watchers contemplate how the cathedral might be improved, micron by micron, and call for the Eaters to come and begin renovations informed by use and necessity (*i.e.*, life-long skeletal development) [7]. As an interconnected web within the skeletal matrix, Cytes sense the mechanical force the cathedral is subjected to and signal plans for remodeling to account for demands place upon the structure. These Cyte-derived signals are interpreted by bone lining cells - which likely shift their identity in response and become a precursor cell type (*e.g.*, osteoprogenitor, reversal cell, etc.). These precursors then call out for Clasts to make renovations [8]. In these remodeling projects, Clasts lead by removing that which needs improvement (*e.g.*, a site of damage, frailty, inadequate support, replacement of an ill sentinel, etc.). However, these Clasts do not proceed alone. Rather, their remodeling efforts are in close collaboration with osteoprogenitors, reversal cells, and Blast partners, whom they coordinate with through a series of collaborative methods discussed below. So, in all phases of cathedral building, each leader is no tyrant and relies on essential feedback from its colleagues that together form a coordinated *Multicellular Unit* (MU) [3]. These Clast led remodeling units are found on both the trabecular architecture and within the cortical regions of long bones throughout life, and it is estimated that millions are active along your skeleton at any one time [9]. Moreover, these units collaborate to remove, rebuild, and remontoir a bone site for 6–12 month stents [3]. And so, *each boney cathedral is a life-long experiment*, constantly evolving until laid down in final form. Unfortunately, it seems that when one of the remodeling partners becomes ill, they can dominate the partnership - a failure in coordination that inevitability imperils the cathedral’s integrity (more on that below). And such is our current model of how bony cathedrals and their architects operate, but the Reader would do well to remember that all models are a poor attempt at making sense of reality - who is of course infinitely more intricate. Still, we do like our stories, and they serve us well, so long as we do not cling to them.

### Clast Methods of Communication and Coordination Within the MU

As discussed above, Clasts do not function in isolation, rather, their activity is tightly coordinated within MU through dynamic interactions with their partners. The Clast’s intracellular coordination with its partners is achieved through multiple, interconnected modes of communication, including: direct cell-cell contact, secretion of soluble messengers, release of matrix-embedded signals via resorption, and the packaging and release of extracellular vesicles (EVs). Together, these mechanisms of collaboration ensure the precise coupling of bone resorption to formation - an essential relationship for maintaining life-long skeletal integrity Table 1.

**Table 1:** Molecular mediators of Clast communication and coordination within the MU

Molecule	Type of Interaction	Effect	Reference
RANKL	Direct Cell–Cell / Soluble / EV	Promotes osteoclast differentiation via RANK; regulates osteoclast–osteoblast coupling	[3, 8, 10]
OPG	Direct Cell–Cell / Soluble	Decoy receptor for RANKL; inhibits osteoclastogenesis	[10]
LGR4	Direct Cell–Cell	Suppresses osteoclast differentiation; regulates osteoblast maturation	[11, 12]
EphB2–EphB4	Direct Cell–Cell	Bidirectional signaling; inhibits osteoclasts, promotes osteoblast differentiation	[13, 14]
S1P	Paracrine	Osteoclast-derived S1P recruits osteoblasts; modulates bone formation	[15–17]
HGF	Paracrine / Autocrine	Promotes osteoblast differentiation and osteoclast regulation	[18]
LIF	Paracrine / Autocrine	Enhances osteoblast recruitment and differentiation	[24, 25]
CT-1	Paracrine / Autocrine	Promotes osteoblast mineralization; regulates osteoclast function	[27]
SLIT3	Paracrine	Promotes osteoblast migration; maintains bone mass by enhancing angiogenesis	[28, 30]
TGF-β1	Matrix-Derived	Recruits BMSCs; biphasic effects on osteoblasts and osteoclasts	[32, 34, 35]
IGF-1	Matrix-Derived	Stimulates MSC differentiation into osteoblasts via PI3K–Akt–mTOR	[37]
miR-214 (EV)	EV	Inhibits osteoblast activity by targeting ATF4	[41]
miR-324 (EV)	EV	Promotes MSC osteogenic differentiation via ARHGAP1 / RhoA-ROCK	[42]
MiR-150-5p	EV	Inhibits MSC osteogenic differentiation.	[58]
OC-ABs (apoptotic bodies)	EV	Induce osteoblast differentiation via RANKL reverse signaling, mTOR pathway	[45, 46]
SPP1 (EV cargo)	EV	Activates TGF-β1/SMAD3 in MSCs to drive osteoblast differentiation	[43]
Siglec15–TLR2	EV / Immune modulation	Suppresses CD8⁺ T cell activation; links bone remodeling to immune regulation	[47, 48]

#### Direct Cell-cell Interactions

Within the MU, Clasts operate in close physical and functional association with Blast lineage cells. Direct membrane contacts between Clasts, Blasts, and bone-lining cells enable bidirectional signaling that coordinates Clast differentiation, activity, and lifespan [10, 11]. The RANK-RANKL-OPG axis is perhaps the most essential signaling module in Clast biology. RANKL, predominantly expressed by Blast lineage cells (*e.g.*, Blasts, Cytes, bone-lining cells, and osteoprogenitors), binds to RANK on Clast precursors to drive Clastogenesis and maturation. Dysregulation of RANK-RANKL signaling, particularly RANKL overproduction, is a hallmark of age-related skeletal pathologies like osteoporosis and underpins the development of anti-RANKL biologics (*i.e.*, denosumab) for therapeutic intervention. Although mainly membrane-bound, RANKL can also exist in a soluble form generated through proteolytic cleavage or alternative splicing and is expressed across multiple tissues [12]. Notably, in vivo evidence indicates that the membrane-bound form of RANKL is sufficient for most physiological functions in the skeleton, whereas soluble RANKL plays a more limited, context-dependent role [13, 14]. Interestingly, transgenic overexpression of soluble RANKL has potent pathological effects postnatally, leading to severe bone loss, increased Clast number, and reduced bone strength [15]. Moreover, soluble RANKL has a dose dependent effect on Clast size, multinucleation status, and resorptive capacity [16–18]. Together, these findings indicate that, soluble RANKL can significantly enhance Clast formation and function, driving pathological bone resorption when dysregulated. Clasts also regulate Blast behavior via direct contact. Clast EphB2 binds EphB4 on the Blast plasma membrane and bidirectionally coordinates both cells through direct contact [19]. The EphB2–EphB4 axis suppresses Clast differentiation while promoting Blast activity, via ERK1/2 and RhoA-dependent pathways, ultimately coupling resorption to formation [20]. Collectively, direct, physical interactions establish the existence of a tightly regulated network in the “dance” whereby Clasts and Blasts coordinate behavior in the transition from bone resorption to formation. 

#### Paracrine and Autocrine Signaling

Complementing direct contact, Clasts shape the bone remodeling niche through the secretion of soluble factors - Clastokines - that function in both paracrine and autocrine manners to coordinate bone remodeling. One such clastokine, Sphingosine-1-phosphate (S1P) represents a key coupling factor that enhances Blast recruitment and activity while indirectly promoting Clastogenesis by elevating RANKL and suppressing OPG expression in Blasts via the p38/ERK–COX2–PGE2 signaling pathway [21, 22]. S1P release is tightly regulated by calcitonin, which suppresses S1P release via SPNS2 inhibition [23]. Clasts secrete many other factors that recruit and promote the differentiation of Blasts, including hepatocyte growth factor (HGF), complement component C3a, and afamin [24–27]. Moreover, Clast-derived WNT10B activates canonical β-catenin signaling and directs mesenchymal stem cells toward the Blast lineage [28, 29]. Clasts also release Cytokine-like factors that influence Blasts, including leukemia inhibitory factor (LIF) and cardiotrophin-1 (CT-1) and reinforce Clast-Blast coupling. LIF promotes Blast recruitment and is reduced upon Clast inhibition [30, 31], whereas CT-1 exerts both osteogenic paracrine and Clastogenic autocrine regulatory effects through gp130-mediated signaling [32, 33]. Similarly, SLIT3, a conserved Robo ligand, has emerged as an important regulator of bone remodeling, although its cellular origin remains debated [34–37]. Regardless of source, SLIT3 enhances bone formation and exhibits therapeutic potential in bone loss conditions [34, 35, 37]. Is the production of Clastokines impacted by Clast multinucleation? Unfortunately, this gap in knowledge has yet to be directly assessed, as previous reports have evaluated the systemic (in vivo) or populational (in vitro Clast differentiation) production of these soluble factors. However, a previous report has demonstrated that *Ctsk* loss elevates S1P release, and that this elevated S1P promoted Blast activity [38]. Interestingly, in addition to elevating the number of Clasts in vivo and in vitro, Clasts lacking *Ctsk* appear much larger - although Clast size/multinucleation were not quantified. In summary, Clast-derived soluble factors form a complex signaling network that actively links bone resorption to formation.

#### Communication via Bone Matrix

In addition to producing and secreting their own signals, Clasts indirectly regulate bone remodeling through the release of matrix-embedded factors during bone resorption. The bone matrix serves as a reservoir of growth factors that are liberated in active form, providing spatial and temporal cues for subsequent bone formation.

For example, TGF-β1 is stored in a latent form within the bone matrix and activated during resorption [39]. Once released, active TGF-β1 establishes a gradient that recruits bone mesenchymal stem cells (BMSCs) to resorption sites via SMAD-dependent signaling, thereby coordinating bone resorption with formation [40], however TGF-β1 exerts biphasic effects on Blasts, BMSCs, and Clasts depending on concentration and context [41–43]. Clasts also release IGF-1 from the matrix, which promotes osteoprogenitor differentiation and activates PI3K–Akt–mTOR signaling [44]. Age-related declines in IGF-1 signaling contribute to reduced bone formation and osteoporosis [44, 45]. Thus, matrix-derived signals act as a critical intermediary, translating past resorption events into future bone formation in the maintenance of healthy skeletal physiology throughout life. Moreover, Clast resorption and the release of matrix factors are directly correlated with Clast multinucleation status (see below).

#### Extracellular Vesicles

Adding further complexity, Clasts also communicate via the release of EVs, which transfer membrane protected, bioactive cargo between cells [46]. EVs exhibit functional heterogeneity depending on their origin [47]. Clast-derived EVs enriched in miR-214 suppress Blast activity by targeting ATF4 [47], whereas other cargos (*e.g.*, miR-324 or SPP1) promote osteogenic differentiation [48, 49]. EVs can also regulate Clastogenesis directly, either inhibiting via RANK-containing decoy vesicles or indirectly promoting by driving Blast differentiation via apoptotic bodies and RANKL reverse signaling [50, 51]. Interestingly, reduced Clast derived apoptotic bodies (cell death derived EVs [46]) have been observed in murine models of osteoporosis, and systemic administration of OC-derived apoptotic bodies significantly delays bone loss in ovariectomized (OVX) mice [52]. Importantly, EVs also link bone remodeling to immune regulation. Siglec15-positive Clast EVs suppress CD8⁺ T cell activity via TLR2 interaction, highlighting the role of Clast EVs beyond skeletal biology [53, 54]. Overall, the type, concentration, and timing of EV release determine their function, with precursor-derived vesicles promoting Clastogenesis and mature Clast-derived vesicles primarily supporting bone formation. As with soluble signaling factors, previous reports have not evaluated EV release with respect to Clast size/multinucleation and current data simply reflects populational changes in vesicle release.

Taken together, Clasts emerge not merely as bone-resorbing cells but as central coordinators of the MU. If the Reader is interested in learning more concerning the “other” roles of Clasts in physiology, please consult our Colleagues’ deeper perspectives [55–57].

## Multinucleated Osteoclasts – Syncytia with an appetite

Now that we have discussed the physiological niche Clasts occupy and the manner by which they influence the MU during bone remodeling, we will focus on a narrow aspect of Clast biology - ***multinucleation***. During Clastogenesis, the critical signaling factor RANKL ushers Clast progenitors to (1) exit the cell cycle, (2) find likewise prepared partners, and (3) fuse plasma membranes to form a multinucleated, syncytial cell. Despite its appreciation for many decades, much concerning *why* Clasts are multinucleated is still quite unclear. Here we struggle with questions surrounding the consequences of these multinucleation events underpinning Clast maturation. How does multinucleation impact Clast resorptive behavior? Does perturbed Clast multinucleation elicit pathological changes in the skeleton? How long do Clasts live/operate following multinucleation and are Clast nuclei long-lived or transient residents of the syncytia they call home? Below, we will explore multinucleation’s many impacts on the behavior of syncytial Clasts, their functions and dysfunctions, and highlight lovely gaps in knowledge that leave our field the space to dream. Finally, we will close with an idea that both excites and troubles us, but this will require the Reader’s willingness to tolerate a bit of madness. *What if in their dance of making one from many the Clast’s nuclei do not wholly forget themselves but instead temper their individuality and work together in shared delimitation?* A strange dream to entertain, but could recent findings push us to hold lightly to an alternative model where Clasts operate as a ***syncytial community*** - a cell, yes, and perhaps something more? If the Reader is interested in further discussion of the current understanding of Clast differentiation [59, 60], bone eating (*i.e.*, resorption) [61–63], or the making of one from many (*i.e.*, cell-cell fusion) [64, 65], we recommend consulting previous reviews.

### The Interconnectedness of Clast Multinucleation and Resorptive Capacity

A defining feature of Clasts is their multinucleated nature, forming from many precursors through the process of cell-cell fusion. *The number of nuclei within a multinucleated Clast determines its resorption capacity* [66–68], and Clast resorptive capacity plays a major role in setting the rate of skeletal remodeling [3, 69], regulates the body’s metabolic activity [70], and is essential for human life [71]. The underlying principles connecting Clast fusion, multinucleation, and resorptive activity remain a major gap in Clast biology, however, recent work has clearly illustrated that Clast multinucleation and resorption are deeply interconnected. In healthy physiological conditions, the resorptive capacity and efficiency of a Clast increases as multinucleation increases [67]. Early work by Piper *et al.*, utilizing chick Clasts, revealed that with more nuclei, Clasts created larger and deeper resorption pits in bone [67]. A correlative relationship between Clast multinucleation status and resorptive capacity has also been observed in many other vertebrates [66], including humans, where the number of nuclei per Clast appears to be the best predictor of bone erosion in post-menopausal individuals [68]. Therefore, the regulation of Clast multinucleation is a critical skeletal feature, as it regulates bone volume. Clasts regulate their multinucleation through a network of genes called the macrophage multinucleation network [72], many that are associated with skeletal pathologies, and several previously described proteins [64], however the precise mechanism by which Clast fusion is facilitated in a regulated fashion remains an open gap in the understanding of skeletal biology.

What characteristics of larger, more multinucleated Clasts elevate resorptive function? To resorb, a Clast must first form a sealing zone - the Clast’s resorptive organelle [73]. The sealing zone forms adjacent to the Clast’s characteristic ruffled border and isolates the resorptive machinery that degrades both bone mineral and collagenic matrix [74]. In vitro, average mature Clasts form 2–4 sealing zones whereas smaller Clasts usually make a single sealing zone [75]. Previous reports have demonstrated that “giant” Clasts have the capacity to form up to 8 sealing zones [75, 76]. The increased size and/or number of sealing zones permits larger, more multinucleated Clasts to more efficiently resorb bone, driving the ↑ nuclei = ↑ resorptive capacity paradigm [75]. Part of the relationship between multinucleation and resorptive capacity may lie in a Clast’s transcriptional capacity, where increased nuclear multiplicity elevates the cell’s production capacity of resorptive machinery compared to mononucleated cells [77]. Within a multinucleated Clast, all nuclei are transcriptionally active, but notably, higher transcriptional activity is observed in Clasts associated with resorption pits, illustrating a link between transcriptional and resorptive capacities [78]. Still transcriptional output alone does not explain how a single Clast with 10 nuclei exhibits a higher resorptive capacity when compared to 10 mononucleated Clasts.

Clast motility also impacts erosive behavior within the skeleton. The formation of multiple sealing zones by larger Clasts impacts their motility. As a Clast extends its leading edge to advance, sealing zones form and dissolve bone at the trailing edge [79]. Forward movement requires the dissolution and reassembly of these sealing zones. Recent work has convincingly defined two modes of Clast resorption - pits and trenches [80]. Pits are characterized by a round morphology, stem from stationary resorption, and exhibit increased depth with increased Clast multinucleation [80]. Trenches initiate from pits and are linear extensions of a pit created by Clasts moving across the bone while resorbing [80]. Interestingly, the formation of a continuous trench is reliant on the constant recycling and displacement of the sealing zone assembly [80]. Previous work has characterized Clast “digging” behavior, which strongly correlates with pathology in osteopenic, post-menopausal females [68]. These Clasts displayed high motility during resorption, “digging” trenches rather than localized resorption pits [68]. Trench resorption was found to be the best predictor of osteopenic burden in vivo [68]. Additionally, the number of nuclei per Clast was the best predictor of trench-digging behavior [68]. Despite gaps in the mechanistic understanding of multinucleation’s link to resorptive capacity - more multinucleated Clasts “take bigger bites” out of bone.

### Disease as Teacher

The Argument [Health > Disease] is one we expect will find few contrarians in the realm of medical science. Perhaps this argument is held most closely by our clinical colleagues, for whom the discussion of pathology often involves the act of holding space for those experiencing pain, dysfunction, or the heartache of walking along with another who carries these burdens. We openly recognize that - as individuals who seldom spend time with those who suffer from the diseases we explore - we hold a debt of compassion for those who experience them and our clinical colleagues who care for these siblings. However, with all recognition of our debt of compassion, we will dispense with the view of disease as a problem for solving herein. Instead, we will look to disease as a feature of Evolution - *an ancient Teacher whose lessons offer us the opportunity to better love the bodies that hold “us” and the lovely monsters chewing away at their bones*. After all, it would be an insult to our neighbors who suffer from the pathologies discussed below should we advert our eyes and not learn.

Many pathologies involving a gross change in skeletal mass are associated with elevated Clast number and/or multinucleation. Of note, most of these pathologies are not due to some intrinsic defect in Clasts *per se* but stem from alterations in the bone microenvironment that promote Clastogenesis (*e.g.*, alterations in RANKL, OPG, IL-6, etc.). Beyond referencing such pathologies for their utility in demonstrating the societal value of unraveling the mysteries of multinucleation, can these human models teach us something wonderful about the syncytial communities they distort?

#### Osteoporosis

Osteoporosis is an age-related skeletal fragility distinguished by the systemic loss of bone mass stemming from elevated Clast number, multinucleation status, and resorptive activity [81]. Osteoporosis is most associated with elevated fracture risk, fracture associated pain, and a progressive reduction in stature. Post-menopausal individuals have the highest risk of developing osteoporosis, though the risk increases broadly with age regardless of sex. Using monocytes isolated from post-menopausal, osteoporotic individuals, Moller *et al.*, demonstrated that the fusion capacity of Clasts and their resulting multinucleation status correlate strongly with Clast resorptive capacity, the steady-state level of the fusion promoting protein DC-Stamp, and disease severity [68, 82]. Moreover, increased Clast multinucleation correlated with elevated resorptive metabolites in the post-menopausal donors. In addition to the many systemic changes described in osteoporotic etiology, intracellular ROS signaling in Clasts is elevated in osteoporosis and contributes to elevated Clastogeneisis [83, 84]. Our group has previously show that intracellular ROS also regulates Clast fusion, potentially contributing to elevated Clast multinucleation in osteoporosis [85]. What lessons does osteoporosis have to contribute to the understanding of Clast multinucleation? Likely too many to list herein, however, osteoporosis highlights the impact of systemic changes on the multinucleation status and resorptive capacity of Clasts, independent of Clast specific genetic aberrations. For more, please consult our Colleagues’ deeper considerations of Clasts in osteoporosis [7, 86].

#### Paget’s Disease

Paget’s disease of bone is an osteolytic disorder associated with elevated Clast size and multinucleation stemming from a multifactorial, incompletely understood mechanism. The disorder likely stems from a latent viral infection that stimulates an age-related pathology in genetically venerable populations, most commonly presenting in males [87–90]. While most cases appear spontaneous, the disease can be inherited in an autosomal dominant manner with variable penetrance. Clasts derived from many, if not most, individuals with Paget’s exhibit hallmarks of previous measles infection, express measles virus nucleocapsid encoded transcripts (MVNTs), and measles virus gene transduction imparts a Pagetic phenotype to healthy Clasts [91]. For inherited cases of Paget’s, ~ 40% exhibit mutations in *SQSTM1* - a downstream regulator of RANKL signaling, with mutations in M-CSF and RANKL encoding loci also commonly observed [92]. Knock-in of patient mutations in *SQSTM1* alone are not sufficient to elicit Paget-like lesions in mice, whereas, exogenous expression of MVNTs is. Although, mutations in *SQSTM1* dramatically exacerbate murine Paget-like lesions stemming from MVNT expression [93]. Mechanistically, MVNT expression stimulates IL-6 autocrine signaling in Clasts that elevates IGF1 and exacerbates EphB2–EphB4 between Clasts and Blasts [94]. Although previously considered the second most common metabolic bone disorder [90], more than half of Paget’s cases are asymptomatic - incidentally identified via routine medical screening. Moreover, the incidence of Paget’s is putatively considered to be decreasing with the advent of routine vaccination schedules in countries with well-developed public health programs, a trend tied to the disease’s linkage with paramyxoviral infection [95]. For the young at heart, Paget’s has a long anthropological history, even affecting the skeletons of dinosaurs in the Jurassic period [96]. The disorder itself is associated with elevated Clast multinucleation, increased bone resorption, and compensatory bone formation at single or multiple sites along the skeleton.

What lessons do the mysteries of Paget’s hold? Generally, Paget’s follows a classic paradigm where increased Clast multinucleation = increased resorptive capacity, leading to a net loss in bone mass, pain, and frailty at lesional sites. For something deeper? Paget’s highlights the role of Clasts as a viral harbor along the skeleton [97–99]. Interestingly, viruses that infect Clasts also alter protein transport, reactive oxygen species production, caspase activation, and the posttranslational modifications of host proteins (*e.g.*, altered phosphorylation) [100–102]. We recently identified a regulator of Clast multinucleation - La protein - whose surface trafficking is dependent on reactive oxygen species modification, dephosphorylation, and subapoptotic caspase cleavage [85, 103]. Moreover, La cleavage and altered trafficking have been previously reported in models of polio virus and hepatitis c infection [104, 105] - two viral pathologies associated with osteolytic comorbidities [106, 107]. If Clast multinucleation status is partially regulated by the delivery and temporal retention of cell-cell fusion machinery at the surface of fusion partners, perhaps viral infection and/or antiviral cellular responses alter the retention of Clast fusion machinery at the plasma membrane and drive increased multinucleation and resorptive status in Paget’s. Viruses are the greatest cell biologists after all, having studied cells for many millennia before us. Perhaps these viruses have exploited protein trafficking pathways that, while unfamiliar to us, Clasts specialize in, offering these viruses an advantage in trafficking their own machinery to the cell surface.

#### Osteopetrosis

Osteopetrosis is broadly defined as a pathologic increase in bone density that leads to skeletal frailty and typically presents as one of two subclasses: Clast-rich or Clast-poor [108]. Clast-poor pathologies arise from an absence of Clasts, often attributed to a loss of Clast precursor differentiation capacity [108], while Clast-rich pathologies retain Clasts within their skeletal niche that exhibit defective resorption [109, 110]. The loss of Clast resorptive function stems from a variety missing, functionally essential proteins [111, 112]. This loss results in unbalanced, Blast-dependent bone deposition, and the intricate trabecular network that typically scaffolds long bones loses its mesh-like nature and becomes nearly indistinguishable from solid, cortical bone [113]. Osteopetrosis etiology encompasses a spectrum of symptoms and severities, with autosomal dominant osteopetrosis (ADO) typically manifesting in milder phenotypes and autosomal recessive osteopetrosis (ARO) typically resulting in loss of vision, hearing, and lethality before the age of 10 - also referred to as malignant infantile osteopetrosis [114].

ADO is not typically diagnosed until late childhood or teenage years, while ARO is often diagnosed within a few months after birth, as postnatal development of the skeleton fails [114]. ADO is classified as either ADOI or ADOII. ADOI is characterized by osteosclerosis, increased skull vault density, and no increase in fracture risk in the rest of the skeleton, all driven by a mutation in the LRP5 gene resulting in increased Blast activity [115]. ADOII, the most common form of osteopetrosis, is largely driven by a dominant negative mutation in the chloride channel 7 gene *CLCN7*, resulting in impaired Clast resorption [116]. Recessive forms of osteopetrosis also arise from the dysfunction of Clasts, thus ADOI is widely considered to be mechanistically separated from ADOII and ARO [115]. Patients with ADOII often experience multiple bone fractures, osteoarthritis, osteomyelitis, nerve compression, scoliosis, and spondylolisthesis, among other symptoms [114].

ARO pathophysiology highlights where gaps in the mechanistic understanding of Clast formation and function limit patient care. ARO is caused by recessive, monogenic, loss of function mutations commonly in genes associated with pH regulation and vesicular trafficking - *CLCN7*, *OSTM1*, or *SNX10* - that result in Clast hyperfusion and greatly elevated multinucleation. As established above, larger, mature Clasts exhibit a greater capacity to resorb bone, however, ARO poses an *exception* to the greater multinucleation = greater resorptive capacity dogma. Clasts in *SNX10*-linked ARO fuse to produce syncytia with 10-100x more nuclei that normal physiological states 76. These oversized Clasts do *not* resorb bone, but rather remain in a perpetually immature, fusing state and never transition into a mature resorptive Clasts [113]. While normal, mature Clasts will form multiple, small sealing zones, in ARO Clasts never form a proper sealing zone [110]. The inability to form sealing zones leads to a loss of resorptive capacity manifesting in a near complete loss of catabolic skeletal remodeling required for postnatal skeletal development. This catabolic failure manifests in dense, brittle bones; failure of the bone marrow niche; and loss of vision and hearing via nerve compression [113]. Moreover, loss of function mutations in *OSTM1* [110, 117], *CLCN7* [118], and *SNX10* [119] ultimately lead to death in the first decade of life, as loss of OC activity leads to failed suture remodeling in the skull, impeding postnatal brain development [120].

What lessons do ADO and ARO hold for those of us interested in Clast biology? Perhaps, these pathologies kill the “greater multinucleation = greater resorptive capacity” dogma. Alternatively, they teach us that multinucleation is but a single metric of Clast biology one should consider. Perhaps they demonstrate that syncytial maturation, fusion partner linage, and adhesive capacity must not be ignored. Some may point out that multinucleation means little if a Clast cannot acidify a sealing zone. To them, we would respectfully point out that a Clast does not require a Cl^−^ transporter to form a sealing zone, and yet, in all forms of ARO mentioned, these sealing zones are missing. ADO and ARO hold a secret. If we are to learn their lesson, we must work together and expand our incomplete models of Clast formation and function.

#### Arthritis

Rheumatoid arthritis (RA) - a chronic inflammatory pathology - is commonly associated with inflammation of the synovium, loss of articular cartilage, and loss of subchondral bone. In addition, RA-associated alterations of the skeletal remodeling niche within joints can further lead to mild, systemic bone loss [121]. Many factors are associated with RA-associated bone loss, but elevation of pro-Clastogenic cytokines (*e.g.*, TNF-α, IL-1β, IL-17, and IL-6) [122] and the elevated recruitment of immune cells (*e.g.*, monocytes, macrophages, T-cells, and dendritic cells) [123] play causal roles. In particular, the recruitment of dendritic cells to inflamed joints and their elevated contribution to Clast formation in RA contributes to subchondral bone loss within affected joints [124].

As with many other osteolytic pathologies, RA pathology is associated with increased Clast number, resorptive behavior, and elevated multinucleation. TNF-α signaling alone seems to promote increased precursor fusion and elevated Clast multinucleation [125]. However, peripheral blood mononuclear cells isolated from individuals with RA form an increased number of Clasts that fuse to form larger, more multinucleated syncytia with increased resorptive capacity in vitro - independent of TNF-α application. These cell autonomous differences in mononuclear cells were associated with an increase in the number of CD14^+^ monocytes, but the slight elevation of these precursors seems insufficient to explain the changes in Clast number/multinucleation observed [126].

What lesson does RA have for us? Perhaps RA can serve as a reminder that, while RANKL stands as the essential Clastogenesis factor, a variety of other cytokines seem to modulate Clast formation and function. Perhaps, RA represents an exacerbated model of modulatory Clastogenesis factors poised to deepen our appreciation of soluble regulators of Clast formation and function beyond the RANK-RANKL-OPG axis [127].

#### Fibrous Dysplasia of Bone

Fibrous dysplasia of bone (FD) is caused by mosaic, gain-of-function mutations in *GNAS* leading to an overproduction of RANKL by osteoprogenitors, excessive Clast formation, elevated resorption, and bone frailty at lesional sites along the skeleton [128]. Numerous reports have robustly demonstrated elevated Clast number, resorption, and osteolysis of the skeleton at lesional sites in FD, however, each of these studies primarily relies on histological assessment of TRAP/CTSK positive cells and do not assess the multinucleation status of the syncytia observed [129–131]. We and our colleagues have previously demonstrated that Clast formation is not simply a consequence of FD pathology, but rather, that Clasts play an active role in disease progression by promoting lesional expansion and suppressing the osteogenic differentiation and mineral deposition of osteoprogenitors within FD lesions [132, 133]. However, these studies did not evaluate Clast multinucleation, only number and activity, and our ex vivo [132, 134, 135] models of FD lack adequate controls to decisively assess whether Clast multinucleation is elevated in FD. While Clasts are more abundant and drive disease burden in FD, and we speculate that they are much larger, the multinucleation status of Clasts that reside in FD lesions vs. healthy bone sites in patients with FD remains an open question we hope our clinical colleagues will address in future studies.

#### Osteonecrosis, Osteomyelitis, Periodontal Disease

Osteonecrosis involves the progressive death of bone tissue stemming from inadequate blood flow at specific sites within the skeleton. While rare, osteonecrosis often stems from injury or as a therapeutic complication (*e.g.*, radiation [136], glucocorticoid [137], bisphosphonate [138], anti-RANKL treatments [139], etc.) and typically presents as a medication associated pathology of the mandible or an injury stimulated pathology of the femoral head. Generally, the mandible experiences perhaps the highest levels of remodeling in the skeleton [140, 141] and would be the bone most effected by therapies that suppress skeletal remodeling, while the femoral head is a notable site of biomechanical force, prone to injury [142]. Medication-related osteonecrosis of the mandible results in significant elevation of Clast size, multinucleation, and number, while radiation-related osteonecrosis does not [143, 144]. While Clast number and surface area are also increased at osteonecrotic sites within the femoral head, alterations in size/multinucleation have not been directly described at this site [145, 146].

Osteomyelitis and periodontal disease are both pathologies that typically stem from chronic bacterial infections and lead to progressive osteolysis at infection sites. These sites of osteolysis stem from increased Clast resorptive activity [147, 148]. Previous histological analyses have suggested that, while Clast number and activity are increased in osteomyelitis, syncytial multinucleation status is unchanged [143, 144]. Unfortunately, both previous studies assessed surgical samples from individuals that simply had chronic jaw inflammation - bacterial infection was not confirmed - and the sample number for each study was quite low. In contrast, murine models of osteomyelitis stemming from direct challenge with *S. aureus* have convincingly demonstrated that Clasts become larger, more resorptive, and more multinucleated in osteomyelitis [149]. Moreover, *S. aureus* has been demonstrated to directly infect both Blasts and Clasts, leading to impaired Blast commitment and bone formation vs. exacerbated Clastogenesis and osteolysis, respectively [150–152]. The number of Clasts that form from peripheral blood mononuclear cells isolated from periodontitis patients is greatly elevated [153]. Clast number and area (a reliable indicator of multinucleation status [154]) are also greatly increased in murine models of periodontitis [155]. Moreover, the fusion of labeled Clast precursors is greatly elevated in murine models of periodontitis [156], and inflammatory culture systems, which model limited aspects of periodontal disease, exhibit elevated Clast multinucleation [157].

In osteonecrosis, osteomyelitis, and periodontal disease, perhaps we can glean that there are many methods of frustrating a Clast (*e.g.*, impairing its ability to remodel bone, stimulating it with inflammatory cytokines, or making it sick with bacterial toxin and/or infection). With each, there is a significant chance these Clasts will become larger and more aggressive in their attempts to resorb bone. While lovely, it pays to remember that when frustrated, these monsters “bite” back.

#### Skeletal Metastasis

The skeleton is the third most common site of metastasis, skeletal metastasis is particularly common in prostate and breast cancers, and discovery of metastasis within the bone microenvironment is typically a harbinger of poor prognoses and short survival windows [158]. Often, skeletal metastasis is accompanied by severe pain, impaired mobility, and dramatic osteolysis at metastatic sites. Osteolysis and many of the pathological features of metastatic bone disease stem from increased formation and function of Clasts at sites of metastasis [159]. While Clasts themselves are thought to promote the expansion and survival of metastatic cells within the bone microenvironment [160–163], metastases also have a profound impact on Clast formation, function, and multinucleation. For many cancer types, increased Clast formation and resorption at metastatic sites are well described, yet Clast multinucleation is not specifically evaluated (*e.g.*, metastases originating from the prostate) [164, 165]. However, in metastases originating from the breast or lung, robust modeling indicates that the osteolysis in these malignancies is associated with dramatic elevations of both the size and multinucleation status of local Clasts [166–168]. While the precise mechanisms by which metastasis promotes increased Clast size and multinucleation remain to be determined, general elevation of Clastogenesis factors (*e.g.*, RANKL), inflammatory cytokines (*e.g.*, IL-17), and miRNA-laden extracellular vesicles have all been implicated. Moreover, cancer cells themselves have been observed to fuse with and incorporate into multinucleated Clasts [169, 170]. For a deeper review of the fusogenic properties of cancer cells with unrelated cell types and the therapeutic resistance of the resulting hybrid cells, please consult previous reviews [171, 172].

#### Medical Interventions

While a variety of osteolytic skeletal diseases are associated with elevated Clast multinucleation, number, and activity, pharmacological interventions aimed at suppressing bone resorption can also alter Clast multinucleation. Long-term alendronate application elevates Clast multinucleation, number, and leads to a hyperfusion phenotype associated with increased apoptosis of Clasts with > 20 nuclei [173, 174]. Although the precise mechanisms by which some antiresorptive therapies promote Clast multinucleation remains and open question, Alendronate and other nitrogen containing bisphosphonates are known to inhibit Clast cholesterol biosynthesis and block protein prenylation [175, 176]. Perhaps these off target therapeutic effects are highlighting some role for cholesterol in tuning the biophysical properties of Clast membranes in such a manner that typically limits fusion/multinucleation? Alternatively, Clast’s may simply employ multinucleation in an attempt to compensate for a more challenging matrix to resorb.

### Clast Lifespan and Theseus’ Paradox

In looking further into the effort and care Clasts employ in the process of multinucleation, we became curious as to when a Clast meets its end? Are these bone eaters short-lived syncytial assemblies destined for apoptotic removal following a quick (days-weeks) collagen buffet or long-lived communities that await osteoprogenitor direction for many months or even longer? Previous claims for both possibilities exist.

*Within the following section*,* we will refrain from assuming that Clast nuclei and Clast syncytia are synonymous*,* as we suspect this assumption may have led to a host of misconceptions in the literature. Instead*,* for the following section*,* we will operate within the hypothesis that Clast syncytia are a temporal*,* communal assemblies of Clast nuclei within a shared delimitation -****a syncytial community***.

#### ~ 2 Weeks

A quick search online, query submitted to one’s large language model of choice, or consulting of a variety of reviews would suggest that there is little controversy over the lifespan of Clasts - around 2 weeks [50, 177–179]. Why would we even discuss this question if it is so easily answered? Primarily because we suspect that these claims are based on a misunderstanding that started roughly three decades ago. Follow the tales of short-lived clasts and the Reader will not find experimental evidence cited until they arrive back in 1994 where an evidence-based statement is made - “individual osteoclast ***nuclei*** are short-lived, turning over about 8% per d, replaced by new preosteoclasts that originated in the bone marrow” [180]. It is here that you will find reference to the rigorous work of Jaworski *et al.*, who employed tritiated thymidine to follow the turnover of Clast nuclei at remodeling sites within the ribs of adult beagles. They found that labeled nuclei generally take 2–4 days to incorporate into Clast syncytia and remain readily detectable for ~ 12 days. Because Jaworski *et al.*, did not observe more than 1–2 labeled nuclei within a syncytium, which averaged ~ 9 nuclei each, they interpreted that these labeled syncytial communities were preexistent rather than formed *de novo*. And so, we are aware of no evidence that supports short-lived Clasts in vivo, although there exists a variety of lines of older inquiry that suggests that Clast *nuclei* can be short-lived and that ablation of Clast precursors limits syncytia survival to ~6 weeks. However, these studies seem to demonstrate that Clast syncytia require continual fusion for survival, not that the syncytia themselves die every 6 weeks [181]. The argument that Clast syncytia are short lived assumes that Clast nuclei and the syncytium where they reside are synonymous. The evidence, as we are aware, instead suggests they operate with some independence. Instead, Clast nuclei seem to be temporary visitors that operate in syncytial space. If Clast syncytia live short lifespans, why are apoptotic syncytia not readily observable along the bones of healthy vertebrates? Sure, one can find a nucleus undergoing apparent pycnosis within a Clast syncytium where it resides, but we cannot find an example of an apoptotic Clast syncytium in vivo that has not been poisoned with bisphosphonates, some superphysiological treatment/stimulus, or that does not reside on a bone that is experiencing some gross pathology. If Clasts apoptose as a syncytial community in vivo where are they? We propose, as others have suggested, that Clasts represent a membrane delimited community made up of many nuclear members that are but temporary residents.

#### Many Months

Elegant experiments from Jacome-Galarza *et al*., indirectly support that Clasts are quite long-live [182]. This seminal study primarily focused on resolving the source of Clast precursors throughout development and dissected the independent contributions of erythro-myeloid progenitors as the embryonic source vs. haematopoietic stem cells as the postnatal source of murine Clasts. However, time-course parabiosis experiments within this study strongly support a >6-month lifespan for murine Clast syncytia. Following 4 weeks of parabiosis between *Csf1r*^*cre*^;*Rosa26*^*LSL−YFP*^*and Csf1r*^*cre*^;*Rosa26*^*LSL−tdTomato*^, Jacome-Galarza *et al.*, observed that 87% (±11%) of TRAP^+^ multinucleated Clasts on the surface of the long bones shared both markers. Shockingly, after separating parabionts for 14 weeks there was no loss of doubly labeled Clasts, and roughly 2/3rds remained doubly labeled for at least 6 months. Barring some yet to be described transfer and colonization of long-lived progenitor pools between parabionts, these data are not compatible with short lived Clast syncytia in mice. Moreover, this study found that a simple cellular infusion of monocytes between these animals was sufficient for nuclear incorporation, and that the incorporation of progenitors into Clast syncytia from a single infusion resulted in the maintenance of these nuclei in syncytial community for a minimum of 8 weeks. Edu-labeling experiments in this study suggest that these long-lived Clast syncytia continually incorporate nuclei at a rate of roughly 1 nucleus every 4–8 weeks, even as the syncytial communities themselves maintain a consistent number of nuclei. These data beg for a mechanism of Clast nuclear disposal/recycling. In addition to this study, Yahara *et al.*, demonstrated that EMP precursor labeling at E9.5 results in Clast nuclei that persist in 6-month-old mice [183]. Collectively, these findings give context to the mechanism by which 2-week parabiosis “cured” osteopetrotic mice for at least 10 months described >45 years ago [184]. These parabionts’ Clasts gained functional nuclei to add to their syncytial communities.

#### Theseus’ Paradox

**Fig. 1:**
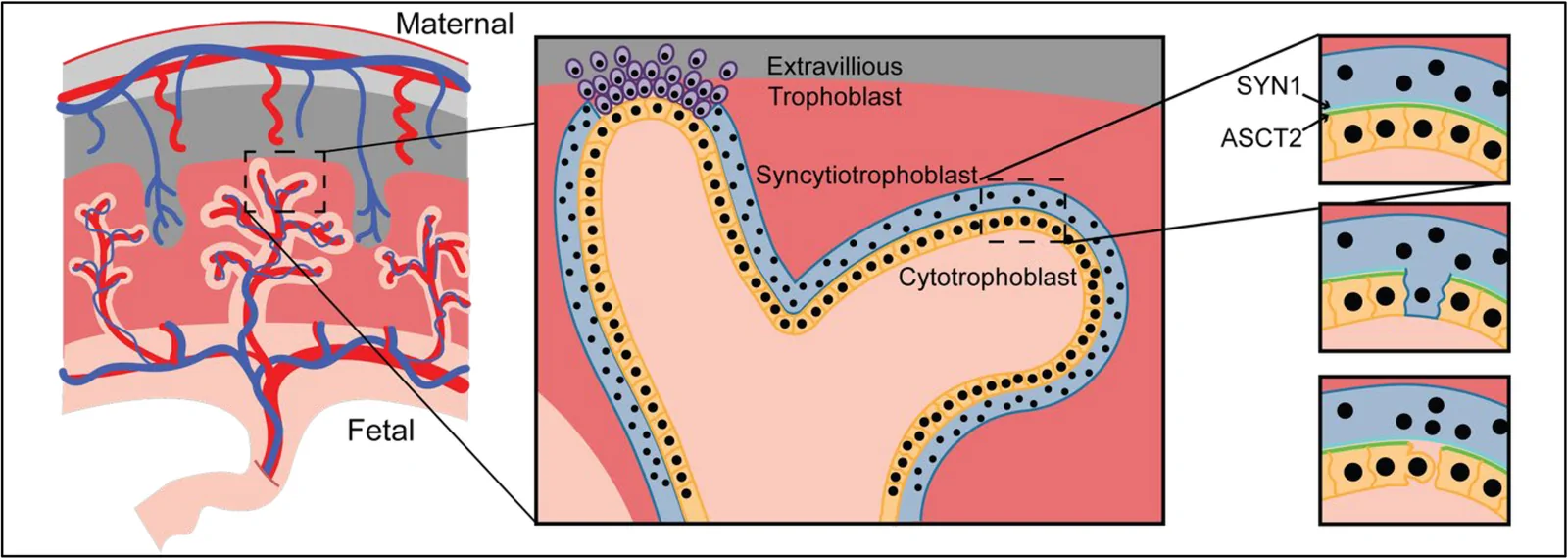
SynT rely on continual fusion for its barrier function in the placenta. (Right) An illustration of the placental villus ultrastructure (grey = maternal, pink = fetal). (Center) An illustration of the trophoblast layer of the placental villi. (Right) An illustration of CytoT fusion with the SynT layer of the placental villus. Syn1 = putative trophoblast fusogen, and ASCT2 is its cognate receptor.

Are Clasts short- or long-lived? Perhaps the skeleton maintains separate pools of short-lived and long-lived Clasts? We would like to suggest a fourth possibility - that the assertions that Clasts live for 2 weeks vs. many months refer to the same Clasts. We expect that data and/or assertions supporting a short Clast lifespan are, in reality, assessing the timeline of an individual community member (*i.e.*, nucleus), where long lifespan refers to the outlook of the community that member resides in (*i.e.*, syncytium). The previous findings discussed above suggest that Clasts behave like other syncytial cell types that are maintained via continual fusion of precursors into their multinucleated community. A community that, in turn, disposes of individual members when they exhaust their lifecycle [185]. The outer covering of the placental villi - the syncytiotrophoblast (SynT) - is a massive syncytial unit that acts as the interface for maternofetal exchange, protects the fetus from immune and pathogen intrusion, and is the source of a multitude of developmental hormones and growth factors [185]. Proper SynT health is essential for a successful pregnancy. Perturbations in the growth, fusion, and continuity of SynTs are commonly associated with ischemic placental diseases [186–189], which account for > 50% of medically indicated preterm births in the U.S [90]. The placental SynT monolayer originates from the fusion of mononucleated cytotrophoblasts (CytoT) from within the fetal villi (Fig. 1) and grows to nearly 60 billion nuclei in one interconnected syncytium at full term [190]. Following initial SynT development, CytoT terminally differentiate and fuse along their apical contact with the SynT throughout pregnancy, continuously providing the SynT with a range of biologically active molecules beyond the need for growth of the SynT’s surface area (Fig. 1) [191]. This continual fusion appears to play a major regulatory function for the SynT layer, as most nuclei in the syncytium have low transcriptional activity, and many consider the mRNA contributed through continual CytoT fusion a requirement for physiological function. What does a SynT do with all the nuclei that come from these constant fusions? Well, they sort the aged nuclei in “knot” structures (*i.e.*, a sort of membrane encapsulated escape pod) and extrude them into the maternal circulation for recycling [192]. So, SynT do not apoptose their community but instead “bury their dead”. Reports suggest that skeletal muscle fibers - the long-lived neighbors of bone - likely also dispose of individual myonuclei. These contractile communities maintain a consistent number of nuclei following their postnatal growth phase (*i.e.*, they can “count”), and evidence suggests that muscle fibers maintain a myonuclear disposal process that recycles a nucleus rather than inflicting a muscle with the need to replace an entire fiber each time a myonucleus is damaged [193–195]. However, the mechanisms underpinning myonuclear disposal are not understood, and as with all new things, these views are not without controversy. The fate of Clast nuclei has been assumed to follow the death of the syncytial community in which it resides, but we would encourage our community to think again concerning the fate of Clast nuclei. One alternative - perhaps syncytial Clasts dispose nuclei similar to SynT? However, why would Clast syncytia need to maintain a nuclear disposal mechanism like SynT if an ill nucleus could simply leave?

Recent work by McDonald *et al. *[196]. has demonstrated Clast syncytia can undergo fission, producing mononucleated Clasts termed “oseomorphs” that leave the bone surface and return to the circulation. McDonald *et al.* are not the only group to observe syncytial fission in Clasts, but the role of fission in physiologic skeletal biology remains *a grand gap in the understanding of life-long skeletal development*. Interestingly, Clasts are not the only post-mitotic syncytial communities capable of fission in vertebrates. Amphibian muscle fibers undergo fission and mitotic-reentry to regenerate skeletal muscle following the loss of an appendage [197, 198]. One can be tempted to dismiss muscle fiber fission as a strategy lost to the evolutionary past, but strikingly, a preparation of amphibian muscle can stimulate mammalian muscle fiber fission and mitotic-reentry [199]. Superphysiologic? Certainly, but this finding demonstrates the machinery for myofiber fission remains intact in mammalian muscle fibers. Now, much debate remains concerning Clast fission, its biological role, and what superphysiologic stimuli one needs to enrich this strange behavior in Clast syncytia to observe enough fission events to study the process. Again, regardless of these debatable topics, Clasts maintain the machinery for fission, and we very much doubt Evolution would maintain this machinery simply for our entertainment.

Why is no one able to regularly observe apoptotic Clast syncytia at remodeling sites? If Clasts disappear across the skeleton a week following α-RANKL application, why are folks not reporting apoptotic Clast syncytia at intermediate timepoints? We seriously doubt that our community is so pitiful at I-spy.

We would pose a question to the Reader, even as we openly recognize that we do not know the answer ourselves. What if McDonald *et al.* discovered how Clast syncytia meet their end in physiologic systems? It is easy enough to speculate that, like MacDonald *et al.*’s application of OPG-fc, anti-RANKL therapies elicits rampant syncytial fission of Clasts across the skeleton. However, what if these superphysiologic stimuli simply promote a process Clast syncytia utilize to remove and recycle nuclei at the end of their lifecycle? Rare events, sure, but if these little monsters carve away at bones for many months or longer, their giving up of the dying may serve a purpose. Like the SynT’s strategy for its knots, these ill osteomorphs could be cleared and recycled via the same angeogenic routes that first caried them to the skeletal remodeling niche - a most civil “burial”. 

## The Syncytial Community Hypothesis

And so, a lovely yet troubling question returns to us as we contemplate Clast death - *what is an osteoclast*? We answered this question above, and yet, the question seems to not be finished with us. We ask that the Reader understand that we openly recognize the following: Clasts are cells, Clasts are multinucleated, and Clasts do not always behave as individual cells.

**Fig. 2:**
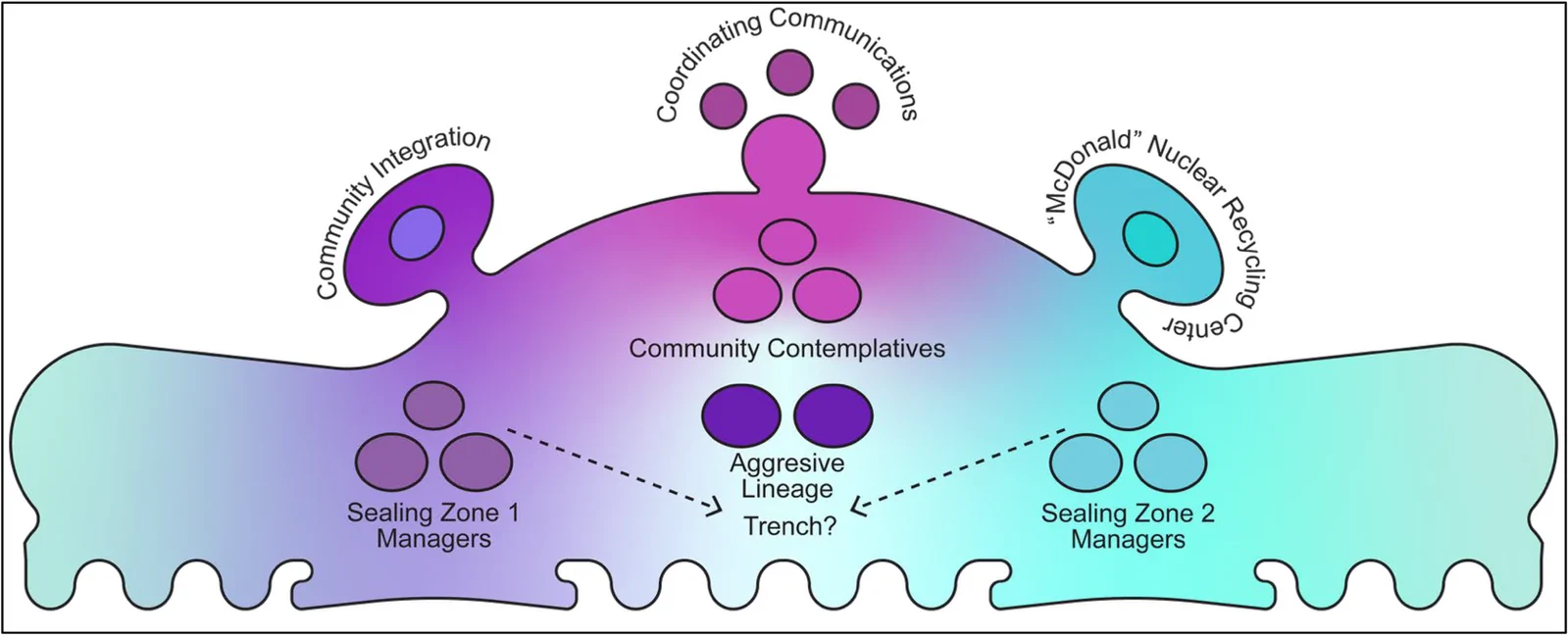
The syncytial community hypothesis: Clast nuclei play specialized functions within a shared cell body. An illustrated Clast undergoing fusion (community integration) and fission (“McDonald” Nuclear Recycling Center). Clusters of coordinated Clast nuclei are depicted actively managing intercellular signaling (Community Contemplatives), maintaining two independent sealing zones, and two nuclei from an inflammatory lineage may recruit others to form a trench

And now, for *a bit of madness*: (1) if Clast nuclei maintain independent lifespans, (2) if Clast syncytia rely on continual fusions/additional nuclei for survival, (3) if, despite continual fusions, Clast syncytia maintain a consistent number of nuclei, (4) if Clast nuclei are free to leave their syncytial residence via fission, and (5) if the number of Clast nuclei can increase such that those nuclei can collaborate and produce additional sealing zones within the same syncytium - are we discussing a *cell* with many nuclei or many *individuals* within a shared border (Fig. 2) - a Syncytial Community?

If Clast behaviors are dictated by the number and perhaps differing originating sources of their many nuclei then syncytial behavior is determined by the syncytium’s “residents”, not the syncytium itself. If a Clast’s many nuclei can leave the syncytial boundaries we assumed contain them, then these nuclei are but temporary residents. Does it make sense to continue thinking of a Clast as an individual cell, like its mononucleated collaborators, or do these bone eaters demand another category? We have a sneaking suspicion that it may be productive to consider the hypothesis that Clasts are not just individual cells but also communities with a shared plasma membrane. In the end, we speculate that the nuclei within a Clast do not wholly forget themselves, despite their shared living arrangements. What a lovely dream - that Evolution, when faced with a great challenge in metazoan biology, would “see” the limits of an individual and instead assemble a community. Perhaps this is the Clast’s greatest lesson - when faced with a challenge at our limits, we need not proceed alone.

## Key References

- Jacome-Galarza CE, Percin GI, Muller JT, Mass E, Lazarov T, Eitler J, Rauner M, Yadav, VK, Crozet L, Bohm M, Loyher PL. Karsenty G, Waskow C, and Geissmann F. Developmental origin, functional maintenance and genetic rescue of osteoclasts. *Nature*. Apr 2019;568(7753):541–545. https://doi.org/10.1038/s41586-019-1105-7
  - ○  Foundational study experimentally demonstrating the long-lived nature of Clast syncytia compared to the continual cycling of Clast nuclei. While this manuscript was published more than 5 years ago, the perspective Veis and O’Brien have share more recently61, concerning the parabiosis experiments’ comment on Clast lifespan, have given this story a new life.
- Delaisse JM, Andersen TL, Kristensen HB, Jensen PR, Andreasen CM, Soe K. Re-thinking the bone remodeling cycle mechanism and the origin of bone loss. *Bone*. Dec 2020;141:115628. https://doi.org/10.1016/j.bone.2020.115628
  - ○  An important synthesis of new findings extending the field’s understanding of bone remodeling. In particular, this perspective highlights the roles of bone lining and reversal cells in the coordinated process of remodeling the skeleton, and the role of osteocytes in delegating RANKL signaling to a third party that in turn recruits Clasts.
- McDonald MM, … Phan TG. Osteoclasts recycle via osteomorphs during RANKL-stimulated bone resorption. *Cell*. Mar 4 2021;184(5):1330–1347 e13. https://doi.org/10.1016/j.cell.2021.02.002
  - ○  Many questions remain concerning the role of Clast fission in healthy skeletal physiology. However, from our perspective, there is no question that McDonald et al., reignited our field’s immagination as to what Clasts are, how they behave, and where they meet their end.
- Reuven N, Winograd-Katz S, Barnea-Zohar M, Pri-Or A, Levin Y, Vacher J, Geiger B, and Elson A. The molecular and functional interplay between the osteopetrosis-associated proteins SNX10, OSTM1, and CLC-7 during mouse osteoclastogenesis. J Bone Miner Res. Dec 18 2025. https://doi.org/10.1093/jbmr/zjaf196
  - ○  Some would disagree, but from our perspective, Reuven et al., have shifted the paradigm of understanding in the pathophysiology of autosomal-recesive osteopetrosis, from a disease of lost acidification competence to a sort of Clast “Peter Pan” syndrome - where Clasts continually fuse, grow, and never mature. Of course Clasts in autosomal-recesive osteopetrosis still do not acidify a sealing zone, but we expect that this seminal publication will serve as a catalysit for uncovering the mechanism that underpins osteopetrotic Clast pathology.

## Data Availability

No datasets were generated or analysed during the current study.
